# The Influence of Work Engagement on Employee Affect and Creativity: Insights from Occupational Mental Health

**DOI:** 10.3390/bs14121217

**Published:** 2024-12-18

**Authors:** Lan Ye, Yanwei Li, Na Zhang, Jian Zhang

**Affiliations:** 1 College of Cabin Crew, Civil Aviation University of China, Tianjin 300300, China; 2 School of Economics and Management, Civil Aviation University of China, Tianjin 300300, China; 3 School of Economics and Management, Beijing Information Science and Technology University, Beijing 102206, China; 4 School of Economics and Management, University of Science and Technology Beijing, Beijing 100083, China

**Keywords:** creativity, affect, work engagement, occupational mental health

## Abstract

Affect plays a pivotal role in shaping employees’ work performance and mental health, with growing recognition of its capacity to drive creativity. However, the differential impacts of positive and negative affect on creative performance remain a subject of debate. This study aims to compare the relationships between high- and low-arousal affect, as well as PANA dimensions of affect, and creative performance, emphasizing the mediating role of work engagement from an occupational mental health perspective. A survey was conducted involving 278 employees and their managers across 25 companies in China. The findings reveal significant associations between high-arousal positive affect, low-arousal positive affect, high-arousal negative affect, and low-arousal negative affect with both work engagement and creative performance. Moreover, the results indicate that work engagement partially mediates the relationship between high-arousal affect and creative performance while fully mediating the relationship between low-arousal affect and creativity. These findings underscore the importance of occupational mental health—particularly work engagement—in fostering employee creativity and highlight its critical role in organizational management strategies.

## 1. Introduction

In today’s era of digitization and globalization, fostering creativity and innovation among employees is increasingly recognized as a fundamental requirement for enterprises [1]. Companies that lack innovation and creativity often struggle to remain competitive in the market. While high-level employee performance is undoubtedly valuable, it alone is insufficient to secure a competitive edge; cultivating creative behaviors and inventiveness is essential [2]. Employee creativity refers to an individual’s ability to generate novel ideas, insights, or products with scientific, aesthetic, social, or technological value, as acknowledged by experts [3,4]. Creativity, assessed through the twin criteria of novelty and usefulness, is an advanced cognitive activity demonstrated by individuals engaged in the creative process [5]. Therefore, enhancing employee creativity has become a central focus in the fields of management and organizational behavior.

Among the various variables predicting employee creativity, affect is a widely acknowledged yet controversial factor [6,7]. Research has demonstrated that affect plays a crucial role in cognitive processes, with the assertion that “learning does not occur without emotional arousal” [8]. The process of generating creative performance is both shaped by emotional experiences and contributes to complex emotional states [9]. The impact of affect on creativity has been further validated through both theoretical frameworks and empirical studies on creative performance [10]. Currently, affective dimension theory and basic affect theory serve as the primary theoretical frameworks through which scholars explore how affect influences creativity.

However, two key issues regarding the relationship between affect and creativity have yet to yield consistent results. The first central controversy concerns whether positive or negative affect more effectively facilitates creativity [6,7,11]. Some studies suggest that positive affect enhances creativity. For instance, Abele’s research indicates that individuals experiencing positive moods tend to demonstrate greater cognitive fluency compared to those in negative moods [12]. Conversely, other studies argue that negative emotional states can foster greater creativity than positive or neutral emotions [13,14]. Despite these contributions, the literature has yet to reach a consensus on the precise nature of the affect-creativity relationship, suggesting the need for a deeper exploration of contextual and individual factors that may influence these dynamics.

The second unresolved issue centers on the mechanisms through which affect impacts creativity. While motivation is often highlighted as the primary pathway connecting affect and creative performance [15], empirical evidence remains inconclusive. For instance, some studies emphasize the mediating role of intrinsic motivation, while others point to cognitive flexibility or divergent thinking as key processes. Furthermore, emerging research has highlighted the potential importance of additional mediators, such as work engagement, but these findings have not been systematically integrated into existing theoretical frameworks [16]. To address these gaps, it is imperative to examine alternative mechanisms that may explain how affect influences creative processes. This approach would contribute to a more comprehensive understanding of the interplay between affective states, contextual factors, and creative [17].

To address these research gaps, we investigated how affect influences creative performance through the lens of Self-Determination Theory (SDT) [18]. SDT is a well-established and widely applied theory in the fields of motivation and psychology [19]. According to SDT, individuals have three core psychological needs: competence, relatedness, and autonomy [18]. Affect, in this framework, can either facilitate or impede an individual’s efforts to satisfy these fundamental psychological needs, and it can predict whether a person will experience positive or negative psychological [18,20]. Importantly, SDT posits that affect itself is neither inherently positive nor negative; rather, it serves as valuable feedback that provides insight into an individual’s well-being and motivation [18]. While most studies categorize affect into positive and negative dimensions, this approach does not fully capture the complexity of emotional information. Watson and Tellegen offered a more nuanced understanding by dividing emotional experiences into varimax-rotated components, such as positive and negative affect, and unrotated dimensions, including arousal levels [21]. This comprehensive view of affective structure provides valuable insights into its role in creative performance. Accordingly, this study, drawing on Self-Determination Theory, will employ Watson and Tellegen’s classification of arousal levels to assess whether categorizing affect into high- and low-arousal dimensions leads to stable and reliable predictions of creativity.

Second, we propose that work engagement, as a key indicator of employees’ occupational mental health, serves as a mechanism through which both positive and negative affect influence creative performance. Over the past two decades, the field of occupational mental health has seen significant growth, as evidenced by numerous studies, high-profile conferences, journal special issues, workshops on a wide range of topics, and the expansion of employee assistance programs provided by large corporations, hospitals, schools, and universities [22]. Traditionally, the focus of occupational mental health has been on disease and poor health outcomes. However, with the emergence of positive psychology in the early 21st century, there has been an increased emphasis on human potential and optimal functioning [23]. As a result, the focus of occupational mental health has shifted toward promoting the positive aspects of employees’ health and well-being. Occupational mental health is a multidimensional construct, encompassing a broad array of indicators such as exhaustion [24], burnout [25,26], job satisfaction/dissatisfaction [24,26,27], work stress [25], turnover intentions [26], and work engagement [24], among others. Work engagement, which is considered the opposite of workplace burnout, is one of the positive traits in this domain [28]. Defined as a cognitive motivational state characterized by vigor, dedication, and absorption [28], work engagement is viewed as a valuable psychological phenomenon due to its significance for both personal well-being and performance outcomes [29,30]. Research has shown that work engagement positively impacts employees’ health, well-being, and performance [31,32,33]. Furthermore, substantial empirical evidence and theoretical consensus suggest a strong relationship between affect and job motivation [34,35]. Positive job-related emotions, such as pleasure and excitement, are associated with high levels of work engagement [36]. In contrast, negative affect is incompatible with task focus, energy at work, or immersion in ongoing activities [37]. To examine the impact of affect on creativity, particularly the relationship between high- and low-arousal affect and creative performance, this study will measure work engagement as a primary indicator of employees’ occupational mental health in the following sections.

## 2. Literature Review and Hypotheses

### 2.1. Creativity and Affect

In today’s rapidly evolving and competitive landscape, the importance of fostering employee creativity cannot be overstated. Creative employees bring fresh perspectives, innovative solutions, and novel ideas, driving organizations toward growth and success. Consequently, nurturing and harnessing employees’ creative potential has become a strategic priority for businesses aiming to thrive in today’s dynamic and unpredictable environment. In recent years, the significance of affect in the workplace has garnered increasing attention from organizations, as they recognize its critical role in influencing employee performance and well-being.

Affect is one of the most frequently used and least debated measures of creative performance, given its significant role in driving innovation in the workplace [38]. The term “affect” refers to a person’s subjective reaction to external stimuli, encompassing physiological arousal, subjective experience, and outward behavior [39]. Watson and Tellegen categorized affective experiences into varimax-rotated components, such as positive and negative affect, and unrotated dimensions, such as arousal level [21]. This comprehensive understanding of affective structure provides valuable insights into how affect influences creative output. In recent years, the role of affect in creative performance has gained increased attention, partly due to the “affective revolution” [6,7].

Systematic empirical studies have demonstrated a strong relationship between employees’ affect and their work performance, particularly regarding the influence of positive or negative affect on creativity [40]. However, there is a lack of consensus on the differing effects of positive versus negative affect on creative performance. While some studies suggest that positive affect enhances creativity and negative affect hinders it, others provide conflicting evidence. For instance, Isen found that positive affective states stimulate greater creativity compared to negative affective states [41]. Agnoli et al. reported that positive emotions, coupled with a high openness trait, contribute to better creative performance in virtual settings [42]. Chuang et al. highlighted that individuals who frequently engage in helping behaviors at work may better manage or offset the effects of negative affect, leading to higher job satisfaction and enhanced creative performance [43].

Conversely, other studies present empirical evidence that negative affect may contribute more to creative performance than positive or neutral affect. For example, fear has been shown to enhance creativity [44], and negative emotions such as disappointment and guilt can positively influence learning and knowledge acquisition, benefiting creative performance [45]. Some studies suggest that both positive and negative emotions can enhance creative performance at different stages of the creative process. Kaufmann and Vosburg observed that, in certain situations—such as market decision-making—individuals experiencing negative affect are capable of generating solutions with highly creative insights, while those with positive affect often make superficial decisions lacking creativity [46]. Roskes examined how both external and internal constraints can influence creative performance, either positively or negatively, depending on motivational factors [47]. Through a series of studies and theoretical perspectives, Russ discussed how positive and negative emotions contribute to different aspects of creativity, emphasizing the importance of playfulness as a mode of thinking that fosters flexible and imaginative approaches [48].

According to Self-Determination Theory (SDT), affect is neither inherently positive nor negative but rather serves as a valuable source of feedback [18]. SDT posits that affect can either facilitate or hinder an individual’s efforts to satisfy three fundamental psychological needs: competence, relatedness, and autonomy. Moreover, affect plays a critical role in predicting whether individuals will experience positive or negative psychological outcomes [18,20]. The theory further emphasizes that need fulfillment is vital for fostering growth, development, and positive affect, which in turn positively influences creative performance. Conversely, need frustration is associated with negative affect, illness, and psychological pathology [49,50]. Thus, the satisfaction or frustration of these core psychological needs profoundly shapes affective experiences and their subsequent impact on creativity.

Some studies suggest that affective arousal levels may play a more critical role than valence (positive vs. negative) in influencing creativity, as highlighted in Watson and Tellegen’s classification of affect [35,40,51]. However, the findings remain inconclusive. Martindale and Greenough emphasize the pivotal role of emotional arousal in the affect-creativity relationship [52]. Meanwhile, Amabile proposes the possibility of a U-shaped relationship, wherein extreme levels of positive or negative affect enhance creativity, while moderate levels may fail to sufficiently activate the cognitive resources required for creative output [53]. Conversely, James et al. argue for an inverted U-shaped relationship, positing that moderate levels of affect maximize creativity [54]. Despite the well-documented link between affect and creativity, the exact nature of this relationship remains ambiguous.

In response to the limitations of previous measurement scales, the psychology research group at the University of Rochester revised the PANAS, expanding its dimensions to include high-arousal positive affect, high-arousal negative affect, low-arousal positive affect, and low-arousal negative affect [55]. Adopting this expanded classification framework and drawing upon the principles of Self-Determination Theory, this study proposes the following hypothesis:

**Hypothesis** **1.** *High-arousal affect will be positively related to creative performance, while low-arousal affect will be negatively related to creative performance.*

### 2.2. Mediation Effect of Work Engagement

Work engagement is generally defined as a stable cognitive state exhibited by employees who are psychologically present and deeply focused on their tasks [28]. It can also be described as an individual’s psychological presence and attentiveness at work [29]. According to Schaufeli et al., work engagement is a positive, fulfilling, and enduring cognitive state characterized by vigor, dedication, and absorption [28]. Furthermore, Rothbard identifies absorption and attention as two key components of work engagement [29].

Role Investment Theory (RIT), a theoretical framework for understanding employee engagement primarily developed by Stets and Burke [56], posits that individuals allocate time, effort, and attention to roles that fulfill their needs for self-actualization, self-esteem, and social validation. According to RIT, the degree of commitment to a role is shaped by the rewards it provides, including psychological satisfaction, social recognition, and emotional fulfillment. Employees are more likely to engage deeply in their work roles when these roles align with their sense of identity and offer positive reinforcement through both intrinsic and extrinsic rewards. This increased investment of cognitive and emotional resources not only enhances their performance but also promotes overall well-being [56]. Consequently, this theory underscores that work engagement extends beyond task completion, encompassing the psychological and emotional benefits employees derive from their roles.

Additionally, Role Investment Theory emphasizes the pivotal role of affect in the investment process. Positive job-related emotions, such as excitement and enjoyment, are indicative of high levels of engagement and investment in work roles, as they enhance motivation and foster a greater allocation of cognitive and emotional resources [28]. Conversely, negative affect may undermine commitment by diminishing motivation and focus, thereby reducing the energy devoted to the role [37]. This alignment between role-related affect and role commitment is essential for understanding how individuals allocate their energy and attention to work-related tasks.

Based on this theoretical foundation, we propose the following hypothesis:

**Hypothesis** **2.** *High-arousal affect will be positively related to work engagement, whereas low-arousal affect will be negatively related to work engagement.*

Building on Role Investment Theory (RIT), previous studies have highlighted the mediating role of work engagement in organizational outcomes [57]. Work engagement, characterized by vigor, dedication, and absorption, reflects employees’ psychological and emotional investment in their roles. Research suggests that when employees experience high levels of engagement, they are more likely to exhibit positive affect and channel their energy toward creative tasks, leading to enhanced performance [58]. Conversely, employees experiencing negative affect, such as dissatisfaction or fear, tend to have lower levels of engagement and are less inclined to focus on creative endeavors.

Empirical evidence further demonstrates that work engagement mediates the relationship between employees’ affective states and performance outcomes. For instance, work engagement has been identified as a mediating mechanism linking both obsessive and harmonious passion to employee performance [59]. This indicates that the degree to which employees are psychologically immersed in their work significantly influences how affect translates into creative outputs.

Given these findings, we posit that work engagement acts as a critical pathway through which both high-arousal and low-arousal affect impact creative performance. High-arousal affect, such as excitement or enthusiasm, is likely to boost engagement, which in turn fosters creativity. Conversely, low-arousal affect, such as boredom or apathy, may diminish engagement and subsequently hinder creative performance.

Therefore, we propose the following hypotheses:

**Hypothesis** **3a.** *Work engagement mediates the relationship between high-arousal affect and creative performance.*

**Hypothesis** **3b.** *Work engagement mediates the relationship between low-arousal affect and creative performance.*

**Figure 1:**
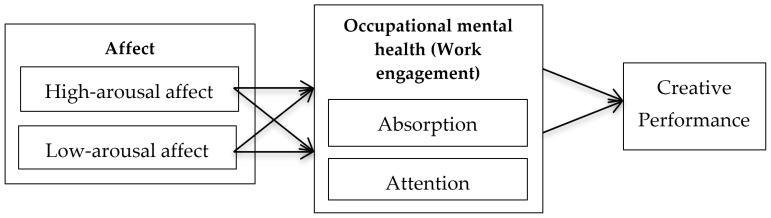
Conceptual model of the study.

The full theoretical model is presented in Figure 1.

## 3. Materials and Methods

### 3.1. Research Design and Sample

The survey was conducted across 25 companies located in Mainland China, involving a total of 278 employees (162 men and 116 women) based in Beijing. Among the participants, 39 individuals (14%) had not completed a college degree, 119 (43%) held a bachelor’s degree, 116 (42%) had attained a master’s degree, and 4 (1%) possessed a doctoral degree.

Participants were grouped into four age categories: 15–30 years, 31–40 years, 41–50 years, and 51 years and above. Regarding tenure, the majority (213 participants, or 77%) had less than ten years of service, 50 participants (18%) had between ten and twenty years of service, and 15 participants (5%) reported more than twenty years of service.

This demographic data provide a comprehensive overview of the participants’ educational backgrounds, age distribution, and professional tenure, ensuring a diverse sample that captures a range of experiences and perspectives. A detailed breakdown of these sample characteristics is presented in Table 1.

### 3.2. Procedure

Employees participated in the study by completing questionnaires that assessed their affect, work engagement, and demographic information, all based on their experiences and emotions during the preceding week. Simultaneously, corresponding managers completed evaluations of their subordinates’ creative performance, focusing on outcomes from the same timeframe. Participants provided informed consent before completing the surveys and were assured that they could withdraw at any point if they felt uncomfortable. Upon completing the surveys, participants were thanked with small tokens of appreciation.

All measurements were conducted in Chinese. The original scales for affect, work engagement, and creative performance were developed in English. To ensure linguistic and conceptual equivalence, a back-translation method was employed to translate the items into Chinese. No significant linguistic discrepancies were identified during this process [60].

### 3.3. Measures

#### 3.3.1. Affect

The study utilized an extended version of the Positive and Negative Affect Scale (PANAS), incorporating 26 affective items to capture a comprehensive range of emotional experiences [55]. Participants rated their responses on a 5-point Likert scale ranging from 1 (never) to 5 (always), based on their feelings and perceptions regarding their work during the previous week. The scale encompassed four distinct categories of affect:

High-Arousal Positive Affect: This dimension included items such as happy, interested, enthusiastic, attentive, alert, and excited, with a reliability coefficient of α = 0.82.

High-Arousal Negative Affect: This dimension measured emotions such as angry, afraid, hostile, jittery, guilty, and anxious, yielding a reliability coefficient of α = 0.87.

Low-Arousal Positive Affect: Items included at ease, relaxed, peaceful, hopeful, grateful, calm, and content, with a reliability coefficient of α = 0.82.

Low-Arousal Negative Affect: This category captured emotions such as bored, sad, disappointed, lonely, sluggish, sleepy, and depressed, with a reliability coefficient of α = 0.86.

By distinguishing between high-arousal and low-arousal affect dimensions, the scale provided nuanced insights into employees’ emotional states and their potential impact on work-related behaviors. The reliability of high-arousal affect is 0.80, and the reliability of low-arousal affect is 0.79. The high reliability scores across all affective dimensions underscore the robustness of the scale in measuring diverse emotional constructs.

#### 3.3.2. Creativity

Creativity was assessed using supervisor evaluations based on the Creativity Performance Measure (CPM) [10]. Supervisors rated each employee’s creative performance across 13 items designed to capture various aspects of creativity in the workplace. A sample item from the CPM is, “Suggests new ways to increase quality”. Responses were scored on a 5-point Likert scale, ranging from 1 (not at all characteristic) to 5 (very characteristic), reflecting the extent to which each item described the employee’s behavior. The measure demonstrated strong internal reliability in this study, with a Cronbach’s alpha of 0.93, indicating high consistency across the items.

#### 3.3.3. Work Engagement

Work engagement was assessed using two key factors developed and tested by Rothbard [29]. Employees completed questionnaires to evaluate their level of work engagement based on their emotional experiences from the previous week. The first factor, absorption, was measured with five items on a 7-point Likert scale (e.g., “I often get carried away by what I am working on.”), yielding a reliability coefficient of 0.85. The second factor, attention, was assessed with four items (e.g., “I focus a great deal of attention on my work.”), which demonstrated a reliability of 0.86. Overall, the total scale reliability for work engagement in this study was 0.91, indicating strong internal consistency.

## 4. Results

Table 2 presents the means, standard deviations, and correlations for the key variables. The results indicate positive relationships between creative performance and low-arousal positive affect (r = 0.24, *p* < 0.01), high-arousal affect (r = 0.21, *p* < 0.01), and high-arousal positive affect (r = 0.32, *p* < 0.01). Conversely, creative performance showed negative correlations with low-arousal negative affect (r = −0.22, *p* < 0.01) and low-arousal affect (r = −0.13, *p* < 0.05). Additionally, work engagement was negatively correlated with low-arousal negative affect (r = −0.24, *p* < 0.01), low-arousal positive affect (r = −0.18, *p* < 0.01), and high-arousal affect (r = −0.14, *p* < 0.05), as well as with high-arousal negative affect (r = −0.21, *p* < 0.01) and low-arousal negative affect (r = −0.24, *p* < 0.01). Furthermore, a positive correlation was found between work engagement and creative performance (r = 0.17, *p* < 0.01). These findings provide support for Hypotheses 1 and 2.

We employed Hayes’s analytical framework [61] to investigate whether work engagement mediates the relationship between high-arousal affect and creative performance. Specifically, Model 4 was utilized, with creative performance (Y) represented by the total score for variable A, high-arousal affect (X) represented by the total score for variable C, and work engagement (M) represented by the total score for variable E. Age, gender, education level, and work tenure were included as control variables to account for their potential influence.

In the first step, PROCESS was used to test the model with work engagement as the outcome variable. The regression coefficient *a* = 0.16 was significant (*p* < 0.01), indicating that high-arousal affect is positively associated with work engagement. In the second step, PROCESS tested the model with creative performance as the outcome variable. The regression coefficient *b* = 0.18 was significant (*p* < 0.01), suggesting a positive relationship between high-arousal affect and creative performance. In the third step, the regression coefficient *c* = 0.16 remained significant (*p* < 0.01) even after including work engagement in the model. This finding indicates that work engagement serves as a mediator, albeit partially, in the relationship between high-arousal affect and creative performance. Thus, Hypothesis 3a was supported.

Additionally, the PROCESS analysis yielded the following results for the model, as shown in Table 3: a total effect of 0.17, a direct effect of 0.14, and an indirect effect of 0.03. The proportion of the mediating effect was calculated as 0.03/0.17 = 17.6%, with a 95% confidence interval (CI) of (0.02, 0.14), which does not include zero. This finding further confirms the presence of a statistically significant indirect effect. Therefore, we conclude that work engagement serves as a partial mediator in this relationship.

Next, we examined whether, similar to high-arousal affect, work engagement mediated the relationship between low-arousal affect and creative performance. This study also employed Model 4 from the PROCESS macro, with Y (Creative Performance) representing the total score for creativity, X (Low-Arousal Affect) representing the total score for low-arousal affect, and M (Work Engagement) representing the total score for work engagement. Age, gender, education level, and years of work experience were included as control variables.

In the first step, the PROCESS tested the model with work engagement as the outcome variable. The regression coefficient *a* was −0.38, which was statistically significant (*p* < 0.01), indicating a negative relationship between low-arousal affect and work engagement. In the second step, the PROCESS macro tested the model with creative performance as the outcome variable. The regression coefficient *b* was −0.62, which was also statistically significant (*p* < 0.01), suggesting a negative relationship between low-arousal affect and creativity. In the third step, the regression coefficient *c* was −0.02 and not statistically significant (*p* > 0.05) after including work engagement as a mediator in the model. This result indicates that work engagement fully mediates the relationship between low-arousal affect and creative performance. Thus, Hypothesis 3b was supported.

Furthermore, the PROCESS generated the following results for the model presented in Table 3: a total effect of −0.56, a direct effect of −0.23, and an indirect effect of −0.15. The proportion of the mediating effect was calculated as −0.15/−0.56 = 26.8%, with a 95% confidence interval (CI) of (0.06, 0.09), which does not include zero. This result provides additional evidence for the presence of an indirect effect. Accordingly, we conclude that the mediation effect is statistically significant, with work engagement serving as a full mediator in this relationship.

To examine the effects of high- and low-arousal affect, as well as positive and negative affect, on creativity and to explore differences in their mediating mechanisms, we further employed the previously described the PROCESS approach to analyze the mediating role of work engagement in the relationship between affect and creative performance (Table 4). The findings revealed that work engagement partially mediated the relationship between positive affect and creative performance. In contrast, no significant mediating effect was identified in the relationship between negative affect and creative performance.

To better understand and compare the effects of the two dimensions—high- versus low-arousal levels and positive versus negative affect—on creative performance, as well as the mediating role of work engagement, we continued to employ the PROCESS Model 4 to analyze the mediating effects of work engagement on the relationships between high-arousal positive affect, high-arousal negative affect, low-arousal positive affect, low-arousal negative affect, and creativity. As shown in Table 5, the results indicated that work engagement partially mediated the relationship between high-arousal positive affect and creativity. However, no mediating effect of work engagement was observed between high-arousal negative affect and creativity. For low-arousal affect, work engagement fully mediated the relationship between low-arousal positive affect and creativity, while it partially mediated the relationship between low-arousal negative affect and creative performance.

## 5. Discussion

Understanding employees’ emotions, thoughts, and affective experiences in the workplace is crucial for managers seeking to implement effective strategies to enhance creative performance [3]. Although earlier studies have provided valuable insights into the impact of affect on creativity [62], inconsistencies persist in findings regarding the relationship between affect and creative performance. Through the critical lens of work engagement as a key indicator of occupational mental health, our study investigates how affect influences creativity, validating the arousal perspective and offering clarity to prior research.

Our findings highlight several important mechanisms. High-arousal positive affect, such as enthusiasm and excitement, fosters creativity by energizing cognitive and emotional resources needed for innovative thinking. In contrast, low-arousal affect—both positive (e.g., calmness) and negative (e.g., boredom)—was found to have a more complex relationship with creativity, with low-arousal negative affect exerting a particularly detrimental effect. These results align with the dual-process model, which posits that high-arousal states are more conducive to divergent thinking, while low-arousal states may hinder cognitive engagement.

Moreover, work engagement was identified as a crucial mediator in the affect–creativity relationship. Employees with higher levels of engagement were better able to translate their emotional states into creative outcomes, providing empirical support for the role of engagement in occupational mental health and performance. This mediating role not only elucidates the pathways through which affect influences creativity but also provides a framework for addressing inconsistencies in earlier findings.

### 5.1. Theoretical Contributions

This study makes several significant theoretical contributions. First, by distinguishing between high- and low-arousal affective states, our research validates the arousal perspective on affect. This nuanced understanding extends prior findings by illustrating how varying levels of arousal interact with workplace dynamics to influence creative performance. Second, our findings expand Self-Determination Theory by demonstrating how affect, categorized along the dimensions of valence and arousal, satisfies psychological needs such as autonomy, competence, and relatedness. These fulfilled needs, in turn, enhance both work engagement and creativity. Third, the study advances Role Investment Theory by providing empirical evidence on how affect shapes cognitive and emotional investments in work roles, ultimately fostering creativity. This underscores the intricate interplay between affective experiences, engagement, and role-based performance. Finally, while previous research has predominantly emphasized intrinsic motivation as the central mediator between affect and creativity, our study identifies work engagement as an additional mechanism. This broader theoretical framework integrates emotional, motivational, and behavioral factors, offering a more comprehensive understanding of the affect–creativity relationship.

### 5.2. Practical Implications

From a practical perspective, our findings provide actionable strategies for organizational leaders seeking to enhance creative performance. First, fostering high-arousal positive affect, such as enthusiasm and excitement, is essential. Managers can create environments that stimulate these emotions through recognition programs, team celebrations, and setting challenging but achievable goals that inspire motivation and creativity. Second, while high-arousal positive affect is critical, balancing affective states is equally important. Managers should also promote low-arousal positive affect, such as calmness, which supports emotional stability and reflective problem-solving. Initiatives like mindfulness training and flexible work arrangements can help achieve this balance. Third, organizations should consider incorporating affect assessments into recruitment and employee development processes. Evaluating candidates’ emotional profiles and distinguishing between high- and low-arousal tendencies can optimize team composition for creative performance. Finally, promoting work engagement is vital, as it emerged as a pivotal mediator in our study. Managers can enhance engagement by aligning roles with employees’ intrinsic interests, providing autonomy, and offering opportunities for professional growth. These strategies collectively create a workplace conducive to both engagement and creativity.

### 5.3. Limitations and Future Research

Despite its contributions, this study is not without limitations. First, it examined 26 types of emotional content using the PANAS, which may not fully capture the complexity of workplace affect. Future research could explore discrete emotions or pairs of discrete emotions to understand their differential impacts on work engagement and creativity. This approach would address the limitations of broad emotional classifications and enrich theoretical contributions by uncovering the unique pathways through which specific emotions influence creative performance. Additionally, incorporating mixed emotions could further illuminate the nuanced interplay of affective dynamics in organizational contexts. Second, the data were collected within a single cultural context (Mainland China), which may limit the generalizability of the findings. Cross-cultural studies are needed to validate the applicability of the arousal perspective across diverse organizational settings. Third, the reliance on self-reported measures for affect and work engagement, alongside supervisor-rated creativity, may introduce biases. Future research could address this limitation by employing objective performance metrics and multisource feedback to enhance validity. Fourth, while this study identified work engagement as a mediator, it did not examine potential moderators such as personality traits, leadership styles, or organizational climate. Investigating these factors could provide deeper insights into the contextual influences on the affect-creativity link. Lastly, the cross-sectional nature of the study precludes causal inferences. Longitudinal or experimental designs would better capture the dynamic interactions between affect, engagement, and creativity over time.

## 6. Conclusions

In summary, our study empirically validates the arousal perspective on affect and establishes work engagement as a critical mediator in the affect–creativity relationship. By differentiating between high- and low-arousal states, our findings provide a more nuanced understanding of how emotions influence creative outcomes in workplace settings.

These results not only contribute to theoretical advancements in organizational behavior and affective science but also offer practical recommendations for fostering innovation within teams. By promoting positive emotional states, enhancing work engagement, and addressing barriers to creativity, managers can cultivate environments that unlock the creative potential of their employees, driving innovation and organizational success.

## Figures and Tables

**Table 1:** Demographic characteristics.

Demographics	Classification	Frequency	Percent	Cumulative Percent
Age (years)	<31	176	63.3	63.3
	31–40	85	30.6	93.9
	41–50	12	4.3	98.2
	>51	5	1.8	100.0
Gender	Male	162	58.3	58.3
	Female	116	41.7	100.0
Education	Junior college	39	14.0	14.0
	Bachelor’s degree	119	42.8	56.8
	Master’s degree	116	41.7	98.6
	Doctoral degree	4	1.4	100.0
Job tenure (years)	<10	213	76.6	76.6
	10–20	50	18.0	94.6
	>20	15	5.4	100.0

Note. N = 278.

**Table 2:** Descriptive statistics and correlation coefficient.

Variables	M	SD	1	2	3	4	5	6	7	8	9	10	11	12	13	14
1. Age	1.45	0.66	1													
2. Gender	1.42	0.49	−0.11	1												
3. Education	2.31	0.72	−0.33 **	−0.011	1											
4. Job tenure	1.28	0.55	0.82 **	−0.08	−0.38 **	1										
5. High-arousal positive affect	22.41	7.01	−0.04	−0.12 *	−0.01	0.04	1									
6. High-arousal negative affect	11.01	5.68	−0.01	−0.16 **	−0.02	0.03	0.15 *	1								
7. Low-arousal positive affect	24.63	6.92	−0.09	−0.06	0.03	−0.05	0.71 **	0.18 **	1							
8. Low-arousal negative affect	14.97	7.24	−0.10	−0.16 **	0.07	−0.06	0.12 *	0.84 **	0.25 **	1						
9. High-arousal affect	33.42	9.64	−0.03	−0.19 **	−0.02	0.04	0.81 **	0.70 **	0.62 **	0.58 **	1					
10. Low-arousal affect	39.59	11.17	−0.11	−0.14 *	0.07	−0.07	0.52 **	0.66 **	0.78 **	0.80 **	0.76 **	1				
11. Attention	21.72	4.36	0.16 **	−0.10	−0.15 *	0.15 *	0.36 **	−0.17 **	0.15 **	−0.20 **	0.16 **	−0.04	1			
12. Absorption	26.22	5.65	0.17 **	−0.08	−0.17 **	0.15 *	0.40 **	−0.21 **	0.19 **	−0.25 **	0.12 *	−0.05	0.84 **	1		
13. Work engagement	47.94	9.62	0.17 **	−0.09	−0.16 **	0.16 **	0.36 **	−0.21 **	0.18 **	−0.24 **	0.14 *	−0.14 *	0.95 **	0.97 **	1	
14. Creative performance	43.31	12.80	−0.04	−0.04	0.18 **	−0.02	0.32 **	−0.05	0.24 **	−0.22 **	0.21 **	−0.13 *	0.15 *	0.15 *	0.17 **	1

N = 278; gender (M = 1, F = 2). ** *p* < 0.01, * *p* < 0.05.

**Table 3:** Mediation Effects of Work Engagement on Affective Arousal Levels and Creative Performance.

Model	Testing Results	R^2^	F	*p*-Value	Total Effect	Indirect Effect	Direct Effect	The Proportion of the Mediating Effect
High-arousal affect => Work engagement => Creative performance	Partial mediation	0.06	6.74	0.00 **	0.17	0.03	0.14	17.6%
Low-arousal affect => Work engagement => Creative performance	Full mediation	0.19	12.10	0.00 **	−0.56	−0.15	−0.23	26.8%

Note. N = 278. ** *p* < 0.01.

**Table 4:** Mediation Effects of Work Engagement on Affective Valence and Creative Performance.

Model	Testing Results	R^2^	F	*p*-Value	Total Effect	Indirect Effect	Direct Effect	The Proportion of the Mediating Effect
Positive affect => Work engagement => Creative performance	Partial mediation	0.09	4.32	0.00 **	0.17	0.02	0.14	11.8%
Negative affect => Work engagement => Creative performance	No mediation	0.06	0.13	0.87	−0.02	0.00	−0.02	0%

Note. N = 278. ** *p* < 0.01.

**Table 5:** Mediation Effects of Work Engagement on Affective Valence, Arousal Levels, and Creative Performance.

Model	Testing Results	R^2^	F	*p*-Value	Total Effect	Indirect Effect	Direct Effect	The Proportion of the Mediating Effect
High-arousal Positive affect => Work engagement => Creative performance	Partial mediation	0.15	4.15	0.00 **	0.13	0.01	0.12	7.7%
High-arousal Negative affect => Work engagement => Creative performance	No mediation	0.04	0.44	0.65	−0.08	0.00	−0.07	0%
Low-arousal Positive affect => Work engagement => Creative performance	Full mediation	0.04	9.39	0.00 **	0.23	0.05	0.13	21.7%
Low-arousal Negative affect => Work engagement => Creative performance	Partial mediation	0.06	7.68	0.00 **	−0.31	−0.06	−0.11	19.4%

Note. N = 278. ** *p* < 0.01.

## Data Availability

The data that support the findings of this study are available from the corresponding author upon reasonable request.

## References

[1] Lu V.N., Wirtz J., Kunz W.H., Paluch S., Gruber T., Martins A., Patterson P.G. (2020). Service robots, customers, and service employees: What can we learn from the academic literature and where are the gaps?. J. Serv. Theory Pract..

[2] Nasifoglu Elidemir S., Ozturen A., Bayighomog S.W. (2020). Innovative behaviors, employee creativity, and sustainable competitive advantage: A moderated mediation. Sustainability.

[3] Amabile T.M. (1996). Creativity in Context: Update to the Social Psychology of Creativity.

[4] Madrid H.P., Patterson M.G. (2016). Creativity at work as a joint function between openness to experience, need for cognition and organizational fairness. Learn. Individ. Differ..

[5] Ding X., Tang Y.Y., Deng Y., Tang R., Posner M.I. (2015). Mood and personality predict improvement in creativity due to meditation training. Learn. Individ. Differ..

[6] Chattopadhyay P., George E., Li J., Gupta V. (2020). Geographical dissimilarity and team member influence: Do emotions experienced in the initial team meeting matter?. Acad. Manag. J..

[7] Conroy S.A., Becker W.J., Menges J.I. (2017). The meaning of my feelings depends on who I am: Work-related identifications shape emotion effects in organizations. Acad. Manag. J..

[8] Weiss P.R. (2000). Emotion and learning. Train. Dev..

[9] Amabile T.M., Barsade S.G., Mueller J.S., Staw B.M. (2005). Affect and creativity at work. Adm. Sci. Q..

[10] George J.M., Zhou J. (2002). Understanding when bad moods foster creativity and good ones don’t: The role of context and clarity of feelings. J. Appl. Psychol..

[11] George J.M., Zhou J. (2007). Dual tuning in a supportive context: Joint contributions of positive mood, negative mood, and supervisory behaviors to employee creativity. Acad. Manag. J..

[12] Abele A. (1992). Positive and negative mood influence on creativity: Evidence for asymmetrical effects. Pol. Psychol. Bull..

[13] Hirt E.R., Melton R.J., McDonald H.E., Harackiewicz J.M. (1996). Processing goals, task interest, and the mood–performance relationship: A mediational analysis. J. Personal. Soc. Psychol..

[14] Martin L.L., Stoner P. (1996). Mood as input: What we think about how we feel determines how we think. Striving and Feeling: Interactions Among Goals, Affect, and Self-Regulation.

[15] Shin S.J., Zhou J. (2003). Transformational leadership, conservation, and creativity: Evidence from Korea. Acad. Manag. J..

[16] Liu W., Li J.W., Zhou Q.W. (2022). Cognitive and social mechanisms: The role of emotions in creativity through work-based learning from a functionalist perspective. Chin. Manag. Stud..

[17] Shalley C.E., Zhou J., Oldham G.R. (2004). The effects of personal and contextual characteristics on creativity: Where should we go from here?. J. Manag..

[18] Deci E.L., Ryan R.M. (2000). The “what” and “why” of goal pursuits: Human needs and the self-determination of behavior. Psychol. Inq..

[19] Hon A.H.Y. (2011). Enhancing employee creativity in the Chinese context: The mediating role of employee self-concordance. Int. J. Hosp. Manag..

[20] Wang N., Zhu J., Dormann C., Song Z., Bakker A.B. (2020). The daily motivators: Positive work events, psychological needs satisfaction, and work engagement. Appl. Psychol..

[21] Watson D., Tellegen A. (1985). Toward a consensual structure of mood. Psychol. Bull..

[22] Jenkins R., Elliott P. (2004). Stressors, burnout and social support: Nurses in acute mental health settings. J. Adv. Nurs..

[23] Shimazu A., Schaufeli W.B. (2008). Work Engagement: An emerging concept in Occupational Health Psychology. BioSci. Trends.

[24] Oksana B. (2018). Professional well-being of practicing physicians: The roles of autonomy, competence, and relatedness. Healthcare.

[25] Fowler K.L. (2006). The relations between personality characteristics, work environment, and the professional well-being of music therapists. J. Music Ther..

[26] Munn E.K., Berber C.E., Fritz J.J. (1996). Factors affecting the professional well-being of child life specialists. Children’s Health Care.

[27] Moshe Z., Dafna H. (2014). Some individual difference predictors of professional well-being and satisfaction of health professionals. Personal. Individ. Differ..

[28] Schaufeli W.B., Salanova M., Gonzalez-Roma V., Bakker A.B. (2002). The measurement of burnout and engagement: A confirmatory factor analytic approach. J. Happiness Stud..

[29] Rothbard N.P. (2001). Enriching or depleting? The dynamics of engagement in work and family roles. Adm. Sci. Q..

[30] Salanova M., Agut S., Piero J.M. (2005). Linking organizational resources and work engagement to employee performance and customer loyalty: The mediation of service climate. J. Appl. Psychol..

[31] Goering E., Shimazu A., Zhou F., Wada T., Sakai R. (2017). Not if, but how they differ: A meta-analytic test of the nomological networks of burnout and engagement. Burn Res..

[32] Neuber L., Englitz C., Schulte N., Forthmann B., Holling H. (2022). How work engagement relates to performance and absenteeism: A meta-analysis. Eur. J. Work Organ. Psychol..

[33] Xanthopoulou D., Bakker A.B., Meyer J.P., Schneider B. (2021). Antecedents and consequences of work engagement: A multilevel nomological net. A Research Agenda for Employee Engagement in a Changing World of Work.

[34] Carver C.S., Scheier M.F. (1990). Origins and functions of positive and negative affect: A control-process view. Psychol. Rev..

[35] Ilies R., Judge T.A. (2005). Goal regulation across time: The effect of feedback and affect. J. Appl. Psychol..

[36] Macey W.H., Schneider B. (2008). The meaning of employee engagement. Ind. Organ. Psychol..

[37] Bledow R., Schmitt A., Frese M., Kühnel J. (2011). The Affective Shift Model of Work Engagement. J. Appl. Psychol..

[38] Mumford M.D. (2003). Where have we been, where are we going? Taking stock in creativity research. Creat. Res. J..

[39] Bledow R., Rosing K., Frese M. (2013). A dynamic perspective on affect and creativity. Acad. Manag. J..

[40] Zenasni F., Lubart T.I. (2008). Emotion-related traits moderate the impact of emotional state on creative performances. J. Individ. Differ..

[41] Isen A.M. (1999). On the relationship between affect and creative problem solving. Affect, Creative Experience, and Psychological Adjustment.

[42] Agnoli S., Zenari S., Mastria S., Corazza G.E. (2021). How do you feel in virtual environments? The role of emotions and openness trait over creative performance. Creat. Theor. Res. Appl..

[43] Chuang Y., Chiang H., Lin A. (2019). Helping behaviors convert negative affect into job satisfaction and creative performance: The moderating role of work competence. Pers. Rev..

[44] Benoit I.D., Miller E.G. (2022). Enhancing creativity perception through fear. J. Bus. Res..

[45] Liu W., Xiang S. (2018). The positive impact of guilt: How and when feedback affect employee learning in the workplace. Leadersh. Organ. Dev. J..

[46] Kaufmann G., Vosburg S.K. (2002). The effects of mood on early and late idea production. Creat. Res. J..

[47] Roskes M. (2015). Constraints that help or hinder creative performance: A motivational approach. Creat. Innov. Manag..

[48] Russ S.W. (2013). Affect and Creativity: The Role of Affect and Play in the Creative Process.

[49] Toyama H., Upadyaya K., Salmela-Aro K. (2022). Job crafting and well-being among school principals: The role of basic psychological need satisfaction and frustration. Eur. Manag. J..

[50] Vansteenkiste M., Ryan R.M. (2013). On psychological growth and vulnerability: Basic psychological need satisfaction and need frustration as a unifying principle. J. Psychother. Integr..

[51] De Dreu C.K., Baas M., Nijstad B.A. (2008). Hedonic tone and activation level in the mood-creativity link: Toward a dual pathway to creativity model. J. Personal. Soc. Psychol..

[52] Martindale C., Greenough J. (1973). The differential effect of increased arousal on creative and intellectual performance. J. Genet. Psychol..

[53] Amabile T.M. (1988). A model of creativity and innovation in organizations. Res. Organ. Behavior..

[54] James K., Brodersen M., Eisenberg J. (2004). Workplace affect and workplace creativity: A review and preliminary model. Hum. Perform..

[55] Watson D., Clark L.A., Tellegen A. (1988). Development and validation of brief measures of positive and negative affect: The PNANS scales. J. Personal. Soc. Psychol..

[56] Stets J.E., Burke P.J. (2000). Identity theory and social identity theory. Soc. Psychol. Q..

[57] Aboramadan M. (2020). The effect of green HRM on employee green behaviors in higher education: The mediating mechanism of green work engagement. Int. J. Organ. Anal..

[58] Sonnentag S. (2003). Recovery, work engagement, and proactive behavior: A new look at the interface between nonwork and work. J. Appl. Psychol..

[59] Ho V.T., Wong S.-S., Lee C.H. (2011). A tale of passion: Linking job passion and cognitive engagement to employee work performance. J. Manag. Stud..

[60] Brislin R.W. (1970). Back-translation for cross-cultural research. J. Cross-Cult. Psychol..

[61] Hayes A.F. (2017). Introduction to Mediation, Moderation, and Conditional Process Analysis: A Regression-Based Approach.

[62] Binnewies C., Wörnlein S.C. (2011). What makes a creative day? A diary study on the interplay between affect, job stressors, and job control. J. Organ. Behav..

